# Maize pollen is an important allergen in occupationally exposed workers

**DOI:** 10.1186/1745-6673-6-32

**Published:** 2011-12-13

**Authors:** Marcus Oldenburg, Arnd Petersen, Xaver Baur

**Affiliations:** 1 Institute for Occupational and Maritime Medicine (ZfAM), University of Hamburg, Hamburg State Department for Social Affairs, Family, Health and Consumer Protection, Germany; 2 Clinical and Molecular Allergology, Research Center Borstel, Parkallee 22, D-23845 Borstel, Germany

**Keywords:** cross-reactivity, IgE reactivity, maize pollen, maize pollination, sensitization

## Abstract

**Background:**

The work- or environmental-related type I sensitization to maize pollen is hardly investigated. We sought to determine the prevalence of sensitization to maize pollen among exposed workers and to identify the eliciting allergens.

**Methods:**

In July 2010, 8 out of 11 subjects were examined who were repeatedly exposed to maize pollen by pollinating maize during their work in a biological research department. All 8 filled in a questionnaire and underwent skin prick testing (SPT) and immune-specific analyses.

**Results:**

5 out of the 8 exposed subjects had repeatedly suffered for at least several weeks from rhinitis, 4 from conjunctivitis, 4 from urticaria, and 2 from shortness of breath upon occupational exposure to maize pollen. All symptomatic workers had specific IgE antibodies against maize pollen (CAP class ≥ 1). Interestingly, 4 of the 5 maize pollen-allergic subjects, but none of the 3 asymptomatic exposed workers had IgE antibodies specific for grass pollen. All but one of the maize pollen-allergic subjects had suffered from allergic grass pollen-related symptoms for 6 to 11 years before job-related exposure to maize pollen. Lung function testing was normal in all cases. In immunoblot analyses, the allergenic components could be identified as Zea m 1 and Zea m 13. The reactivity is mostly caused by cross-reactivity to the homologous allergens in temperate grass pollen. Two sera responded to Zea m 3, but interestingly not to the corresponding timothy allergen indicating maize-specific IgE reactivity.

**Conclusion:**

The present data suggest that subjects pollinating maize are at high risk of developing an allergy to maize pollen as a so far underestimated source of occupational allergens. For the screening of patients with suspected maize pollen sensitization, the determination of IgE antibodies specific for maize pollen is suitable.

## Background

Maize belongs to the family of grasses (Poaceae) and is cultivated globally as one of the most important cereal crops worldwide. It is also an allergen source in contemporary nutrition. Allergy to maize is caused by proteins in the kernels. Zea m 14 as a heat-resistant lipid transfer protein (LTP) with a molecular weight of 9 kDa was identified as a major food allergen of maize mediating an immunoglobulin E (IgE) response [1].

Some allergens in the maize kernel are described to also be present in maize pollen. So far, identified allergens of maize pollen are Zea m 1, Zea m 2, Zea m 3, Zea m 12 and Zea m 13. A certain degree of cross-reactivity among members of the family Poaceae can be supposed as many species of grass and maize pollen contain at least the group 1 and 13 grass allergens [2-4]. However, Suphioglu et al. (1993) demonstrated that not all of the antigenic epitopes of group 1 allergens were cross-reactive [5]. Further, the IgE-binding patterns in immunoblot between maize and other grasses differed considerably.

Buczylko et al. (1995) found that out of 56 maize pollen-sensitized children with hay fever symptoms more than half of them were also sensitized to maize seed allergens [6]. The reason for this might be Zea m 13 and homologous proteins which are present in both maize pollen and maize seed [7].

About 90% of grass pollen-sensitized patients show IgE reactivity to group 5 grass pollen allergens. In maize pollen, group 5 allergens were not found [8].

Schubert et al. (2005) demonstrated that 40 of 77 patients positive to a mixed extract of grass and cereal pollens also had a positive skin prick test to maize pollen [9]. Out of the 40 patients, 14 subjects had specific IgE antibodies against grass and rye pollens, and only 2 of the 14 sera also displayed specific IgE to maize pollen. This is probably caused by the lack of a close taxonomic and immunologic relationship between grass/cereal and maize, which belong to the Pooideae and Panicoideae subfamilies, respectively.

Most major allergenic pollens from grasses, weeds and trees are derived from wind-pollinated rather than from insect-pollinated plants. This is true for clinically important pollens from the various geographic regions [10]. Considering the weight of maize pollen grains between 150 and 500 ng (60 to 125 μm in diameter) [11], they should mainly elicit allergic symptoms of the upper airways. However, due to the large weight of maize pollen falling between 50 and 70 m from its source, the urban population is normally not exposed to this pollen, which can explain the low frequency of maize sensitization in the general population [12]. Therefore, maize pollen has been regarded as a minor agent for hay fever.

To our knowledge, no study investigated the sensitizing potency of maize pollen among workers during maize pollination. The aim of this study was to explore the prevalence of sensitization to maize pollen and to determine whether this is only caused by cross-reactivity. Further, it should be examined whether grass- and maize pollen-specific sensitizations occur with subsequent health risks in a cohort of workers exposed to maize pollen.

## Materials and methods

### Study group

In July 2010, the complete working group of a German biological research department (6 subjects) and 2 of a second working group (with a total of 5 subjects) were examined. Thus, the study group represented 73% of all subjects exposed to maize pollen (n = 11) in that research department. Prior to testing, all subjects were informed about the aim and content of the study and had to give their informed consent for participation. 3 workers refused participation in this study for unknown reasons.

All of the 8 examined workers (6 females, mean age 36.9 years, 2 current smokers) had a history of work-related exposure to maize pollen through repeated maize pollination. At the time of the study, they had been exposed to both wild type maize as well as genetically modified maize for 1.1 to 21.1 years. The duration of pollination lasted from 1 to 5 hours per week and the cumulative exposure to maize pollen - calculated as the product of duration of maize pollination in years and average hours per week - ranged between 1 and 50 years × hours (Table 1). In July 2010, 5 of the 8 subjects were exposed to maize pollen at the time of this study.

**Table 1:** Demographic and exposure data of the subjects participating in the study

	*Subject*							
	1	2	3	4	5	6	7	8
***Male ****(M)****/Female ****(F)*	F	M	F	M	F	F	F	F
*No current Smoker (NS)/Smoker (S)*	NS	NS	NS	S	NS	NS	NS	S
*Pack years*				0.8				4.5
***Cumulative exposure to maize pollen^#^***	29.4	42.2	8.1	7.0	3.2	1.1	50.5	6.0
***Past maize pollination ****(years before this examination)*	0	0	4.1	0	0	0.3	0	0.9
***Use of occupational protection measures***								
***- at first contact with maize pollen***	Dust mask, lab coat	Dust mask, lab coat	Only lab coat	Gloves, headpiece, dust mask	None	None	None	None
- during their past pollination*	Overall, gloves, air-supplied respirator							

(according to work-related symptoms and CAP results (CAP class ≥ 1) subjects 1 to 5 were considered as "maize pollen-allergic")

^#^product of duration of maize pollination in years and average hours per week

*between 2006 and 2007, the use of overalls and air-supplied respirators was introduced at the workplace as an occupational protection measure

### Maize pollination

The ears of the more than 2 m tall maize plants are female inflorescences, tightly covered over by several layers of leaves, with silks at their end as elongated stigmas. The apex of the stem ends in the tassel, an inflorescence of male flowers. When the tassel is mature and conditions are suitably warm and dry, it dehisces and releases pollen. Maize pollen is anemophilous (dispersed by wind) and most pollen grains fall within a few meters of the tassel because of its high settling velocity.

In the investigated biological research department, maize pollination took place in a greenhouse within 3 major steps:

1. A bag is carefully placed over the plant's tassels.

2. The bag is tapped several times to release pollen from the tassels. (This must be done carefully to avoid pollen contamination of the ambient air).

3. The bag is placed above the fresh silk and slightly tapped so that the pollen is deposited onto the silk.

At the beginning of the work-related maize pollination, 4 workers of the research department only used a paper dust mask and/or a lab coat during pollination (Table 1). 4 subjects did not use airway protection. Due to allergic symptoms in 5 workers during pollination, protective overalls and air-supplied respirators (dustmaster 3 M, P2 filters, St. Paul, Minnesota, USA) were introduced at the worksite between 2006 and 2007. An instruction manual described the use of these occupational safety measures during maize pollination in the greenhouse.

### Questionnaire

By means of a standardized questionnaire, demographic data, the current and past exposure to maize pollen during pollination, acute and chronic symptoms of the airways, eyes, and of the skin were recorded. The questions on symptoms were in most parts identical to the questions of the German National Health Interview and Examination Survey 1997/98 (BGS 99) [13]. Allergic symptoms were defined as repeated rhinitis, conjunctivitis, urticaria or shortness of breath for at least several weeks during the past 12 months.

Moreover, the current and former use of available occupational protection measures during maize pollination was recorded. In addition, before and directly after 15 min maize pollination in the greenhouse we used a pre- and post-exposure questionnaire focusing on the subjects' complaints during testing.

### Allergological tests

All 8 workers underwent blood sampling for measurement of IgE to maize pollen and timothy grass pollen as well as for its recombinant allergens Phl p 1 and Phl p 5 by means of UniCAP fluoroenzyme immunoassay (FEIA). Subjects with IgE levels above 0.35 kUA/L (CAP class ≥ 1) and with work-related symptoms were defined as "maize pollen-allergic".

Further, trained assistant medical technicians performed skin prick testing on the volar side of the subjects' forearms with a standardized 1 mm pricker (ALK, Hörsholm, Denmark). The mean wheal size was recorded after 15 min. The subjects were tested with a panel of 22 common commercially available allergenic extracts (*Dermatophagoides farinae, Dermatophagoides pteronyssinus, Aspergillus fumigatus, Cladosporium herbarum, Alternaria alternata, Artemisia, Ambrosia, Parietaria, Platanus*, pollen of early-, mid- and late-blooming trees, grass pollen mixtures, maize kernel, rye, nettle, goosefoot, rape, plantain, animal dander (dog and cat) and latex), as well as a commercially available extract of maize pollen (Bencard Allergie, Munich, Germany). Subjects with at least two positive skin test responses to the panel of 22 common allergens used (with the exclusion of maize pollen extract) were considered atopic.

### Western blotting

Serum samples of the 8 workers were also studied by means of immunoblot analysis. Additionally, sera from healthy individuals and grass pollen-allergic patients were used as controls. Three monoclonal antibodies directed against the allergen grass groups 1, 5 and 13 of timothy grass pollen and a rabbit antiserum directed against Phl p 2/3 served as markers [4].

Lyophilized pollen extracts of maize or timothy grass were separated by SDS-PAGE under reducing conditions as described by Petersen et al. (2006) [4]. Briefly, samples were loaded at a concentration of 18 μg/cm onto homogenous gels (T = 15%, C = 2.6%). After running the gels, the proteins were transferred to nitrocellulose membrane (PROTRAN BA 83, Sigma-Aldrich, Taufkirchen, Germany) by semi-dry blotting at 2 mA/cm^2 ^for 30 min. Molecular mass was determined by the Unstained Protein Molecular Weight Marker (Fermentas, St. Leon-Rot, Germany). For protein staining, strips of the membrane were stained with India ink [14]. For immunodetection, the nitrocellulose membranes were blocked with TBST (0.1 M Tris-buffered saline (TBS), pH 7.4 containing 0.05% (v/v) Tween 20). The membrane was cut into strips which were incubated with subjects' sera (1:10 in TBST). After washing the strips were incubated with the alkaline phosphatase-conjugated secondary antibody, monoclonal anti-human IgE (1:2000) (Allergopharma, Reinbek, Germany) or goat anti-mouse IgG/M (1:10000) (Dianova, Hamburg, Germany), respectively. Binding was visualized by means of substrate solution containing nitroblue tetrazolium chloride (NBT) and 5-bromo-4-chloro-3-indolyl phosphate potassium salt (BCIP) (Sigma) in 0.1 M TBS, pH 9.5 [15].

### 2-D PAGE, immunoblotting and protein sequencing

2-D PAGE was performed as previously described with slight modifications [16]. Briefly, immobilized pH gradient strips (Novex IPG Zoom Strips; Invitrogen, Groningen, The Netherlands) in a pH range of 3 to 10 were used for separation of 200 μg of pollen extract by isoelectric focusing. Subsequently, SDS-PAGE was carried out in the second dimension (Tris glycine Zoom gels 4-20%; Invitrogen). Molecular masses and pIs were determined by comparison with PageRuler Prestained Protein Ladder (Fermentas) and IEF Marker 3-10, Liquid Mix (Serva, Heidelberg, Germany). For the identification of allergens, proteins were transferred by semi-dry blotting and immunostaining as stated above. For protein staining, blotting was performed onto polyvinylidene difluoride membrane using 10 mM CAPS (N-cyclohexyl-3-aminopropanesulfonic acid) with 10% methanol (pH 11.0) as transfer buffer [17] and stained with Coomassie (Serva). Protein bands were excised and microsequencing was performed using a Procise protein sequencer with on-line PTH amino acid analyser (PE Biosystems, Weiterstadt, Germany).

### Lung function analysis

All 8 subjects underwent lung function testing with a portable spirometer (FlowScreen, Erich Jaeger, Wurzburg, Germany). Subjects were in a sitting position and wore a nose clip.

From at least three forced expiratory spirograms, the forced vital capacity (FVC) and the forced expiratory volume in one second (FEV_1_) of each subject were obtained according to the recommendations of the American Thoracic Society (2005) [18]. The ratio FEV_1_/FVC% was calculated. Lung function reference values used were those from Brandli et al. (2000) [19].

Further, non-specific bronchial hyperresponsiveness (NSBHR) was tested by the stepwise application of methacholine using the Pari Provocation test^®^. The applied dose inducing a drop in FEV_1 _by 20% was defined as PD_20 _FEV_1_. NS BHR was diagnosed when PD_20_FEV_1 _was less than 300 μg methacholine (inhaled cumulative dose) [20]. Further, fraction of exhaled nitric oxide (FeNO) was measured according to ATS criteria by using the analysator CLD-88 sp (ECO Medics, Dürnten, Switzerland) [21]. The FeNO upper limit of normal was 20 ppb. Rhinomanometric measurements were performed with the Flow Screen Pro (Viasys Healthcare, Wurzburg, Germany).

Lung function tests including rhinomanometry were performed before and directly after 15 min pollination in the greenhouse of the research department. Acute changes in airway function (Δ of parameters) were expressed for each subject as a percentage of the value before exposure [22]. A significant rhinometric reaction after the challenge test was defined as a decrease of the nasal flow by more than 50%.

## Results

### Symptoms

According to their history, 5 of the 8 examined subjects suffered from allergic symptoms during occupational exposure to maize pollen (5 from rhinitis, 4 from conjunctivitis, 4 from urticaria and 2 from shortness of breath) (Table 2). 4 of these 5 workers developed work-related symptoms within the first few months of their exposure to maize pollen (only one subject after a latency of 10 years).

**Table 2:** Allergic symptoms and sensitization to maize pollen and common environmental allergens

	*Subject*							
	1	2	3	4	5	6	7	8
***Work-related symptoms ****(during maize pollination),^§ ^yes (+)/no (-)*								
*Rhinitis*	+	+	+	+	+	-	-	-
*Conjunctivitis*	+	+	+	-	+	-	-	-
*Urticaria*	+	+	+	-	+	-	-	-
*Shortness of breath*	-	+	-	-	+	-	-	-
***Latency of allergic symptoms after start of maize pollination ****(months)*	2	120	7	2	1	-	5^+^	-
***Latency of maize pollen allergy after onset of grass pollen allergy ****(years)^$ ^*	10	-°	11	9	6	-	-	-
***Maize pollen***								
*- extract (IgE kU/l)^#^*	**5.48**	**3.69**	**1.32**	**0.70**	**0.49**	-	-	-
*- Western blotting*	+	+	+	-	-	-	-	-
*- Skin prick test, pos (+)/neg (-)*	+	+	-	+	n/a	+	-	-
***Grass pollen***								
*- extract (IgE kU/l)^#^*	**4.45**	**0.38**	**5.61**	**2.55**	-	-	-	-
*- Phl p 1 (IgE kU/l)^#^*	**1.16**	-	**1.49**	**-**	-	-	-	-
*- Phl p 5 (IgE kU/l)^#^*	**1.25**	-	**2.52**	**0.70**	-	-	-	-
*- Western blotting*	+	-	+	-	-	-	-	-
*- Skin prick test, pos (+)/neg (-)*	+	+	+	+	n/a	+	+	-
***Skin prick test with common environ- mental allergens***^*&*^	1;2;3*	1;9*	1;2; 4;6*	1;2;3;4;5 7;8;9; 10;11;12*	n/a	1	1;3*	-
								

(according to work-related symptoms and CAP results (CAP class ≥ 1), subjects 1 to 5 were considered as "maize pollen-allergic")

+ = positive result; - = absent or negative result; n/a = not applicable

^§^the subjects were asked if they had ever suffered from repeated rhinitis, conjunctivitis, urticaria or shortness of breath for at least several weeks due to maize pollination

^+^in this subject, grass and tree pollen (but not maize pollen) caused allergic symptoms

^#^significant IgE antibodies (CAP class ≥ 1; > 0.35 kU/l) are bold

^$^the onset of allergy was based on anamnestic data about first atopy symptoms in connection with exposure to grass pollen or maize pollen

° this subject could not remember having had any allergic symptoms after exposure to grass pollen

^&^1 = grass pollen; 2 = cat dander; 3 = pollen of early-, mid- and late-blooming trees; 4 = *Dermatophagoides farinae*, 5 = *Dermatophagoides pteronyssinus*, 6 = *Alternaria alternata*; 7 = dog dander; 8 = *Ambrosia*; 9 = rye; 10 = nettle; 11 = goosefoot; 12 = plantain

*atopic subject (at least 2 positive skin prick tests to common environmental allergens)

The cumulative exposure to maize pollen (Table 1) was not related to the occurrence of work-related symptoms. None of the subjects reported allergic symptoms after ingestion of maize food. 2 of the examined workers (No 1 and 2) took antihistamines.

During the past 12 months, 6 subjects had noticed allergic symptoms independent of the work-related exposure; one subject (No 7) reported on conjunctivitis and urticaria only due to grass and tree pollen.

### Maize pollen sensitization

All 5 workers with allergic symptoms during maize pollination had IgE antibodies specific for maize pollen with CAP class ≥1 (Table 2). These 5 symptomatic subjects (No 1 to 5) were defined as "maize pollen-allergic". Prick test responses to maize pollen corresponded to the IgE findings in all but 2 cases. 4 of the 5 maize pollen-allergic subjects, but none of the 3 asymptomatic exposed workers had IgE antibodies specific for grass pollen in the CAP assay. In 3 of the maize pollen-allergic individuals we determined a positive reaction to Phl p 5, a major allergen of the temperate grasses, lacking in maize.

4 of the maize pollen-allergic workers and 2 of the non-allergic subjects showed a positive skin prick test result with grass pollen. Concerning the skin prick test responses, all 4 subjects with a positive test result for maize pollen also showed responses to grass pollen, but in 2 subjects (No 3 and 7) with a positive test for grass pollen no corresponding positive skin prick test reaction was found for maize pollen.

The 4 tested maize pollen-allergic subjects were atopic according to their skin prick test responses to common environmental allergens. Additionally, 1 of the 3 workers without maize pollen-induced symptoms was atopic (Table 2). All 5 tested atopic workers stated that they had hay fever symptoms (rhinitis and/or conjunctivitis). With the exception of one (who did not recognize allergic symptoms in connection with grass pollen exposure), all maize pollen-allergic subjects had suffered from grass pollen-related hay fever for 6 to 11 years *before *work-related exposure to maize pollen. Maize pollen sensitization was not related to the cumulative exposure to maize pollen.

Skin prick testing with maize *kernel *produced negative results in all workers.

### Lung function tests

FVC, FEV_1 _and FEV_1_/FVC% (% predicted) were within the normal range in all 8 cases (Table 3).

**Table 3:** Lung function and rhinomanometry

	*Subject*							
	1	2	3	4	5	6	7	8
***FVC ***(%)*	109	99	102	108	n/a	100	123	99
***FEV_1 _***(%)*	106	102	88	95	n/a	87	115	98
***FEV_1_/FVC% ***(%)*	103	106	91	89	n/a	91	101	103
***NS BHR**, yes (+)/no (-)*	-	-	+	+	n/a	-	-	-
***FeNO ****(ppb)*	18.5	20.5	25.2	73.4	n/a	9.7	10.9	10.8
***Δ FEV_1 _after vs. before pollination ****(%)*	+6.2	+2.9	+4.0	-7.8	n/a	+12.1	-1.5	n/a
***Δ nasal flow after vs. before pollination ****(%)*	+72.8	**-56.5**	-8.2	+63.5	n/a	+72.9	+40.2	n/a

(according to work-related symptoms and CAP results (CAP class ≥ 1) subjects 1 to 5 were considered as "maize pollen-allergic")

*data were expressed as predicted values (reference values by Brändli et al. (2000))

NS BHR = non-specific bronchial hyperresponsiveness

significant changes of lung function parameters after pollination are bold

n/a = not applicable

Due to personal reasons, 1 out of the 8 subjects refused the methacholine challenge test. 2 maize pollen-allergic subjects, but none of the non-allergic subjects exhibited NS BHR. FeNO was elevated (> 20 ppb) in 3 out of the 4 tested maize pollen allergic subjects, but in none of the 3 non-allergic ones.

### Workplace challenges

6 of the 8 workers performed lung function testing and rhinomanometry and filled in a questionnaire (concerning their current symptoms) before and directly after 15 min maize pollination. The pollination was carried out under usual work conditions (using occupational protection measures and pollination technique as described above). The 2 workers treated with antihistamines suspended their treatment at least 5 days before this maize pollen provocation test. All subjects were asymptomatic before the workplace challenge.

After maize pollination, one subject (No 2) developed hand and neck urticaria, which subsided after the use of antihistamines. Only this subject also showed a significant decrease of the nasal air flow in rhinomanometry. The other workers remained free of allergic symptoms and did not show major changes of the nasal air flow. Lung function parameters were not impaired (Table 3).

### Immunoblot analyses

For identification of the allergens in maize pollen, Western blotting was performed. Sera of the 8 individuals exposed to maize were investigated on maize and timothy grass pollen extract blotted onto nitrocellulose membrane after SDS-PAGE.

**Figure 1:**
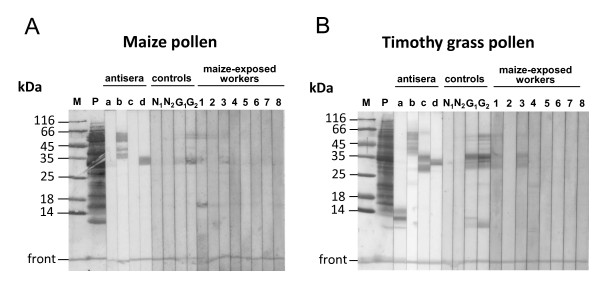
**Determination of IgE reactivity of workers exposed to maize pollen extract (A) and timothy grass pollen extract (B) by means of Western blotting**. M, molecular weight marker. P, protein staining of the pollen extracts with India ink (molecular weight is given according to the marker proteins), a-d, identification of allergens by use of antibodies raised in timothy grass pollen (a, rabbit anti Phl p 2/3; b, anti Phl p 13 (moab AF6); c, anti Phl p 5 (moab Bo1); d, anti Phl p 1 (moab IG12)); N_1_, N_2_, sera of healthy individuals; G_1_, G_2_, sera of grass pollen-allergic patients; 1-8, sera of the 8 workers exposed to maize pollen.

As shown in Figure 1A, the sera of the maize pollen-allergic subjects 1 and 3 recognize a component at approximately 32 kDa (Zea m 1). The protein band is the most prominent protein in the extract. Sera of subjects 1, 2 and 3 bound maize components of 55 kDa, while 1 and 2 additionally recognized a 14 kDa allergen (Zea m 13 and Zea m 3, respectively). For reference, we used monoclonal antibodies (lines b to d) assigning the 32 kDa band to allergen grass group 1 (Zea m 1; line d) and the 55 kDa band to group 13 (Zea m 13; line b). No band is detected by the group 5 specific monoclonal antibody (line c). The antiserum raised against the Phl p 2/3 grass pollen allergens (line a) shows no IgE reactivity with a corresponding protein at about 14 kDa, but a faint binding to the 32 kDa allergen indicating a cross-reactivity between group 2/3 and 1.

For comparison, we determined the IgE reactivity of the maize pollen-exposed workers to timothy grass pollen, a frequent temperate grass species of our region (Figure 1B). IgE-reactive proteins are only detectable in the cases of the maize-exposed subjects 1 and 3 at a molecular range of 35 to 28 kDa. Besides the 32 kDa band identified as Phl p 1 by the monoclonal antibody (line d), these sera additionally recognize proteins of 35 and 28 kDa referring to the group 5 allergens, which are lacking in maize pollen. These results are in accordance with the CAP data for Phl p 5 indicating that these maize pollen-exposed persons are sensitized to grass pollen allergens.

**Figure 2:**
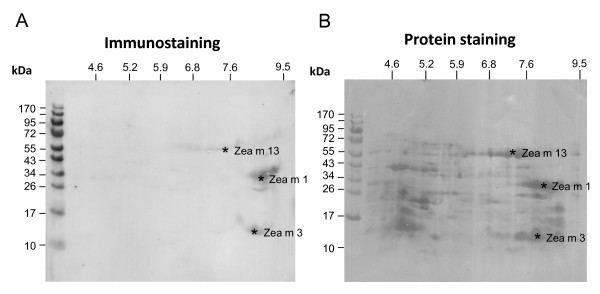
**2D PAGE blotting of maize pollen extract**. (A) IgE immunostaining using serum from subject 1; (B) Protein staining with Coomassie. The marked allergens were identified by monoclonal antibodies and/or protein sequencing.

The most meaningful patient's serum 1 was studied in more detail. Maize pollen extract was separated by 2D PAGE and immunostained for the identification of IgE-reactive components. The immunoblot (Figure 2A) confirms the IgE-reactive protein spots at 14, 32 and a faint reactivity at 55 kDa. The last two proteins were identified as Zea m 1 and Zea m 13 by the monoclonal antibodies, respectively. Since the 14 kDa allergen was neither recognized by the monoclonal antibodies nor by the anti-Phl p 2/3 antiserum (Figure 1A), we excised this protein spot (Figure 2B) and analyzed it by protein sequencing. The N-terminal sequence TTPLTFQVGKGS clearly identified the allergen as Zea m 3 (AY331720). The fact that this allergen was not recognized by the anti-Phl p 2/3 antiserum suggests structural differences between the homologous allergens.

## Discussion

This study focused on the health risks due to maize pollen during the pollination in a biological research department. The examination revealed maize pollen allergy in 5 of 8 examined workers who repeatedly performed maize pollination. All 5 of these workers had CAP class ≥ 1 and suffered from work-related rhinitis during maize pollination. The high weight of maize pollen explains obviously why most of the symptoms in the present study were manifested on the upper airways and only in 2 cases on the lower airways. There was no evidence of an asymptomatic maize sensitization in the other 3 workers. The duration of exposure to maize pollen in total (years) and in hours per week appeared not to be associated with the frequency of maize pollen sensitization. With the exception of one worker, the maize-pollen allergic workers developed allergic symptoms for 1 to 7 months after the onset of maize pollination.

A Spanish study with 101 asthma patients revealed that 57% of the cohort had specific IgE to maize pollen [23]. However, it is not clear whether maize pollen sensitization was due to direct contact with them or due to cross-reactivity with grass pollen.

There is still little knowledge about the clinical relevance of maize pollen in the occupational setting. A recent case history described a 55-year-old person working in a rural area where maize was cultivated in abundance on a large scale [12]. This farmer developed recurrent episodes of rhinoconjunctivitis and asthma in relation to occupational exposure to maize cultures. The documented seasonal pollinosis coincided with the maize pollination. Blood analysis revealed a high IgE antibody level against maize pollen but none against grass pollen. In a further study, Freemann (1994) introduced 6 Navajo patients who had developed respiratory symptoms (sneezing, coughing, and wheezing) due to oral maize pollen used in Navajo ceremonials [24]. In latter ceremonials maize pollen was placed on or under the tongue, or eaten. Some studies suggested that subjects exposed to maize pollen were prone to develop asthma, allergic rhinitis and/or allergic conjunctivitis [23,25,26].

In the present study, all maize pollen-allergic subjects were atopic. This is in line with previous findings that elevated specific IgE and positive skin prick test responses to specific allergens are more pronounced in subjects with allergic manifestations (25%) than in the general population [27,28].

At the beginning of the exposure to maize pollen most of the maize pollen-allergic, but none of the non-allergic workers had already used occupational protection measures during pollination. This is evidence of a pre-employment risk due to cross-reacting sensitization. Maize pollen shares similar allergens with other cereals but notably also with grass pollens. Similar skin prick test responses to grass and maize pollen suggest an antigenic relationship between grass and maize pollen. Petersen et al. (2006) demonstrated that timothy pollen extract completely inhibited IgE binding to maize pollen, whereas maize pollen blocked IgE reactivity to only some timothy pollen allergens [4]. On the basis of inhibition tests, Kalveram et al. (1978) supposed that grass pollen extract contains all antigens typical for maize pollen [29]. On the other hand, some other studies found a low degree of cross-reactivity between grass and maize pollen extracts [30-32].

Performing Western blotting, we could identify IgE reactivities for 3 of the 5 maize pollen allergic individuals. Although immunoblotting is less sensitive than the CAP assay because of protein denaturation, it enables the identification of the allergens. This is important, since a component-resolved diagnostic with single maize pollen allergens is not available.

As reported by Petersen et al. (2006) [4], the structural similarities between the homologous group 1 and 13 allergens of maize and timothy grass pollen reveal sequence identities > 61%. Therefore, cross-reactivities exist between Zea m 1 - Phl p 1 and Zea m 13 - Phl p 13, respectively, however we also identified maize-specific IgE reactivity suggesting different epitopes.

Zea m 3 showed a strong IgE reactivity with one serum. Interestingly, this component was not recognized by the antiserum raised against homologous allergen Phl p 3 in timothy grass pollen and the sequence identity between Zea m 3 and Phl p 3 is only 35% [4]. Thus, it is an example of low structural similarities between homologous allergens of common grasses and maize. Such structural differences can cause maize-specific IgE reactions, although an exclusive maize pollen allergy should be rare because of the lower number of allergen groups in maize and the morphological differences of the pollen compared to the temperate grass species.

In the present study, 4 of the 5 maize pollen-allergic workers had IgE antibodies level > 0.35 kU/l (CAP class ≥ 1) and four had a positive skin prick test to grass pollen. According to their history, all four subjects with allergic respiratory symptoms, both after exposure to grass pollen and maize pollen, observed hay fever symptoms due to grass pollen several years *before *the onset of maize pollen-related symptoms. However, as the sera of only 2 of the 5 maize pollen-allergic workers were positive to group 5 allergens which cause positive reactions in more than 90% of grass pollen-allergic subjects, the molecular pathogenetic path of maize pollen sensitization via grass pollen sensitization remains unclear. In total, our findings suggest that maize pollen sensitization is often associated with grass pollen sensitization, and the IgE reactivity can be largely explained by cross-reactive allergens. But maize-specific IgE reactivities can superpose the existing grass pollen allergy and thus enhance the allergic reactions.

As all subjects with maize pollen-related respiratory symptoms had specific IgE antibodies and two skin prick test responses to maize pollen did not correspond to allergic symptoms, it is supposed that IgE antibodies are more specific for clinically relevant maize pollen sensitization than skin prick tests. Concerning maize food allergy, in a double-blind placebo-controlled study Scibila et al. (2008) displayed a sensitivity of specific IgE levels and skin prick tests of 1.00 and 0.846, respectively [33]. The authors assumed that CAP test with maize may be a suitable test for screening patients with suspected food allergy. More studies are needed to also substantiate this assumption for maize pollen.

None of the investigated workers, including those with maize pollen-induced rhinitis, showed allergic symptoms after ingestion of maize. Although a recent study suggested that foods may play a role in exacerbation and continuance of respiratory manifestations such as allergic rhinitis [27], there was - according to their history - no evidence of a food allergy among the examined workers in this study.

During our current examination, none of the workers showed an obstructive ventilation pattern. However, as a limitation of this study, it is possible that our current examination does not reflect the workers' health status at the onset of exposure to maize pollen several years ago. First, it cannot be excluded that the lung function was impaired initially at the beginning of maize pollination; the improvement of occupational protection (at least since 2007) with a subsequent sufficiently diminished exposure to maize pollen may have resulted in a normalization of lung function. Second, one of the maize pollen-allergic workers and two of the non-allergic workers had had no maize pollen exposure for 4.1, 0.3 and 0.9 years at the time of our examination, which might have led to an attenuated allergic response.

The examination before and after workplace exposure under use of usual occupational protection measures (overall and air-supplied respirator) showed urticaria and rhinitis in one subject but did not reveal impairment of lung function in any worker. This indicated an adequate occupational protection in most of them. Although all workers withdrew the maize pollen which had descended onto the overall to a large extent by suction after pollination, exposure to maize pollen could obviously not completely be avoided; the subject with hand and neck urticaria as well as nasal obstruction after maize pollination was only shortly exposed to pollen while removing the overall. All workers exposed to maize pollen tolerated the use of an air-supplied respirator during maize pollination as an adequate measure for primary prevention.

## Conclusion

Nowadays, only few people are occupationally or environmentally exposed to maize pollen due to the large pollen size and high weight [34]. The obvious causal relationship between maize pollination and respiratory symptoms and the proved sensitization to maize pollen indicates that the examined workers of the biological research department had acquired maize pollen-induced rhinopathy as an occupational disease. The high prevalence of sensitization to maize pollen in the present study emphasizes their strong sensitization potency upon intensive airborne contact. Thus, maize pollen constitutes a potent occupational allergen for directly exposed subjects.

## Ethical Approval

The study was performed with the approval of the Institutional Review Board and is in compliance with the Helsinki Declaration.

## Competing interests

The authors declare that they have no competing interests.

## Authors' contributions

As the project leader MO developed the study design and was responsible for the examination on the spot. He wrote the article and discussed the clinical data. XB gave substantial contributions to conception of the study; especially concerning the lung function testing and the discussion of the allergic findings (Skin-prick-testing, IgE antibodies). AP was responsible for the immunoblot analysis and discussed its findings.

The manuscript has been read and approved by all authors. All of them participated substantially in analysis and discussion of data.
